# Accurate measurement of the Sagnac effect for matter waves

**DOI:** 10.1126/sciadv.abn8009

**Published:** 2022-06-10

**Authors:** Romain Gautier, Mohamed Guessoum, Leonid A. Sidorenkov, Quentin Bouton, Arnaud Landragin, Remi Geiger

**Affiliations:** LNE-SYRTE, Observatoire de Paris-Université PSL, CNRS, Sorbonne Université 61 avenue de l’Observatoire, 75014 Paris, France.

## Abstract

A rotating interferometer with paths that enclose a physical area exhibits a phase shift proportional to this area and to the rotation rate of the frame. Understanding the origin of this so-called Sagnac effect has played a key role in the establishment of the theory of relativity and has pushed for the development of precision optical interferometers. The fundamental importance of the Sagnac effect motivated the realization of experiments to test its validity for waves beyond optical, but precision measurements remained a challenge. Here, we report the accurate test of the Sagnac effect for matter waves, by using a Cesium atom interferometer featuring a geometrical area of 11 cm^2^ and two sensitive axes of measurements. We measure the phase shift induced by Earth’s rotation and find agreement with the theoretical prediction at an accuracy level of 25 parts per million. Beyond the importance for fundamental physics, our work opens practical applications in seismology and geodesy.

## Teaser

The universal effect of rotations on interferometers has been confirmed with extremely high accuracy for matter waves.

## INTRODUCTION

The study of the effect of rotations on interferometers dates back to the late 19th century and is intimately tied to the development of the theory of relativity. In 1913, Georges Sagnac was the first to report an experimental observation of the shift of the fringes in an interferometer subject to a constant rotation rate and its interpretation in the framework of an eather theory (*1*–*3*). Observing the small phase shift induced by Earth’s rotation motivated Michelson, Gale, and Pearson (*4*) to build an interferometer of 0.2-km^2^ area; in 1925, they reported a measurement of the predicted effect with 3% accuracy. The advent of the laser boosted the development of gyroscopes based on the Sagnac effect with the realization of ring laser gyroscopes (*5*) and later of fiber optical gyroscopes (*6*, *7*), which are a key component of modern navigation systems.

The importance of understanding the fundamental nature of the Sagnac effect for the development of modern physics has motivated the realization of rotating interferometers of increasing precision involving other-than-optical waves. Observations were subsequently made with various systems, starting with superconducting electrons (*8*) as one of the first demonstration of a macroscopic matter wave coherence in superconductors. It was followed by measurements with neutral particles: first with neutrons (*9*) and then with thermal atoms (*10*), where the Sagnac effect was found to be in good agreement with theory. A measurement with electron jet (*11*) has extended its validity toward matter waves of free charged particles. Study of the Sagnac effect in superfluid quantum liquids [helium 4 (*12*) and helium 3 (*13*)] and gases [Bose-Enstein Condensate (BEC) (*14*)] has illustrated its universality. These proof-of-principle experiments served to underline the relativistic nature of the Sagnac effect. The first precision measurement was done in 1997, with a reported accuracy of 1% for a thermal matter wave interferometer (*15*). Development of cold atom experiments allowed for measurements of increasing precision (*16*, *17*) up to 0.05% preceding this work.

According to the Sagnac effect, the phase shift in an interferometer of oriented area $\vec{A}$ and subject to a constant rotation rate $\vec{\Omega }$ can be expressed as

$$\Phi_{\Omega }=\frac{4\pi E}{hc^{2}}\vec{A}\cdot \vec{\Omega }$$ (1)

where *E* is the total energy of the interfering particle, and *h* is the Planck’s constant (*E* = *h*ν for photons, *E* ≃ *mc*^2^ for slow massive particles). Precisely testing the validity of this equation requires an accurate knowledge of the interferometer geometry (i.e., of the area vector $\vec{A}$) and of the rotation rate ($\vec{\Omega }$). Exploiting Earth’s rotation, which is known with high accuracy, meets the latter requirement. However, precisely controlling the geometry of a matter wave interferometer of large area (i.e., of high sensitivity) remains a challenge; for example, the accuracy of superfluid helium interferometers has been barely assessed (*18*), while neutron interferometers could test Eq. 1 at best with 0.4% accuracy (*9*).

Cold-atom interferometers feature a high degree of accuracy owing to the good knowledge of the light-matter interaction process exploited to realize the interferometer building blocks, which offers the possibility to quantify the interferometer scale factor using frequency measurements (*19*, *20*). Here, we use a two-axis cold-cesium atom interferometer with a macroscopic area A ≃ 11 cm^2^ (in each direction) rotated by Earth. Our measurements confirm the prediction of Eq. 1 with an accuracy of 25 parts per million (ppm), which represents an improvement of more than 20 compared with previous experiments (*16*, *17*) and allows us to place a constraint on Gödel’s model of a rotating universe (*21*). Moreover, the ability to accurately determine the scale factor of our gyroscope combined with its relative compactness and control of its area orientation [compared with giant ring laser gyroscopes (*22*)] opens practical applications in seismology and geodesy. The configuration of our instrument has advantages over other geometries of cold atom gyroscopes. Its single source folded interferometer rejects accelerations while preventing systematic errors because of trajectory mismatch in twin atom source sensors (*16*, *23*–*25*). Our sensor offers substantial sensitivity gain compared with that of compact atomic gyroscopes (*26*–*28*), allowing us to test the Sagnac effect due to Earth’s rotation with the accuracy level reported in this work.

## RESULTS

**Fig. 1.**
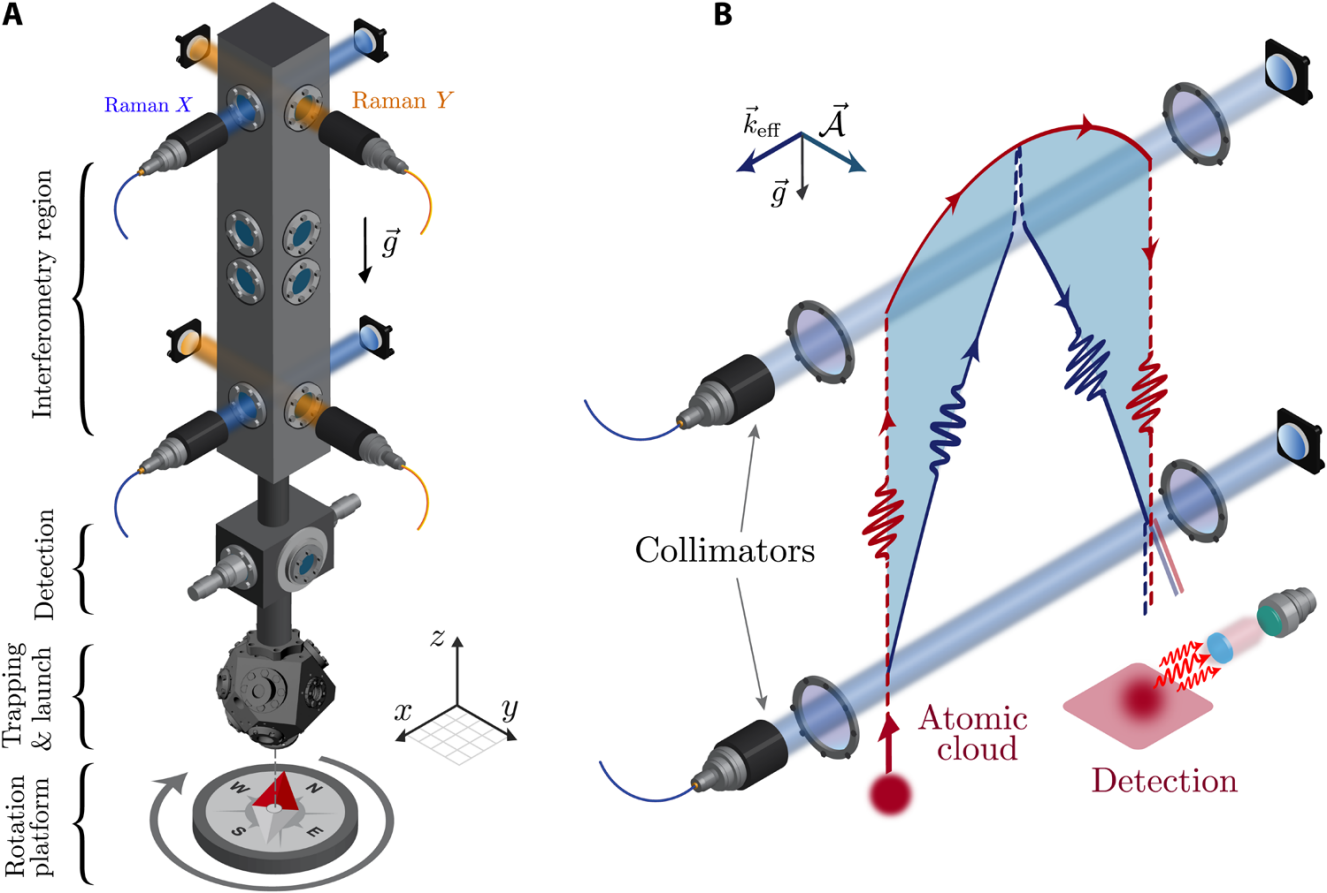
Principle of the experiment. (**A**) Schematic of the sensor head. In the lower part of the vacuum chamber, the cesium atoms are laser cooled and trapped in a magneto-optical trap (MOT), and then launched vertically in the hyperfine state *F* = 4. Subsequently, the atoms enter the interferometer where a sequence of four Raman transitions is driven by retro-reflected laser beams at the top and the bottom of the upper part of the vacuum chamber. The interferometer can be operated either in the *X* (blue beams) or *Y* (orange beams) direction. At the output of the interferometer, the probability for an atom to occupy one of the two internal states *F* = 3 and *F* = 4 is measured by fluorescence detection. The experiment is placed on a rotation stage that allows us to vary the projection of the oriented interferometer area on the Earth rotation vector. (**B**) Schematic of the wave packet propagation in the interferometer (here in the *X* direction, not to scale). The red and blue lines show the two distinct paths of the splitted matter waves enclosing a physical area, underlined by the cyan color. Dashed and plain lines encode the two internal states of the atom.

The core of our experiment and its principle are illustrated in Fig. 1 and have been described in previous works (*29*, *30*) and summarized in Materials and Methods. The interferometric sequence comprises four Raman pulses of Rabi area π/2, π, π, π/2 occurring at times *t* ≃ (0, *T*/2, 3*T*/2, 2*T*), with *T* ≃ 400 ms.

The two-photon Raman transition transfers a momentum $\hbar {\vec{k}}_{\text{eff}}$ to the deflected atom, which, together with the action of gravity acceleration $\vec{g}$ along the path, results in an interferometer area (for perfectly parallel Raman beams)

$$\vec{A}=\frac{T^{3}}{4}\frac{\hbar }{m}{\vec{k}}_{\text{eff}}\times \vec{g}$$ (2)

With the total energy of the interfering atom given by *E* ≃ *mc*^2^ (valid for atoms moving much slower than light), the Sagnac phase shift becomes

$$\Phi_{\Omega }=\frac{T^{3}}{2}({\vec{k}}_{\text{eff}}\times \vec{g})\cdot \vec{\Omega }$$ (3)

The Raman beams are set to an angle θ_0_ with respect to the horizontal plane (perpendicular to $\vec{g}$) to lift the degeneracy associated with the two possible directions of momentum transfer and thereby choose the direction of atom diffraction $\left[\pm {\vec{k}}_{\text{eff}}\right]$. The vector product is then expressed as ${\vec{k}}_{\text{eff}}\times \vec{g}=k_{\text{eff}}g\text{cos}\;(\theta_{0})\vec{n}$, where $\vec{n}$ is a unit vector in the direction of interferometric area ($\vec{A}/|\vec{A}|$) lying in the horizontal plane.

The experiment is placed on a rotation stage, which allows us to change the angle between $\vec{n}$ and the angular velocity of the Earth $\vec{\Omega }$ pointing from south to north. The rotation angle Θ can be varied within 2π with μrad accuracy, thus permitting a precision measurement that is not limited by uncertainty in positioning of the north.

The Sagnac phase shift can therefore be explicitly written in terms of the control parameters as

$$\Phi_{\Omega }(\Theta )=\frac{T^{3}}{2}k_{\text{eff}}g\text{cos}\;(\theta_{0})\times \text{cos}\;(\psi )\Omega_{E}\times \text{cos}\;(\Theta -\Theta_{N})$$ (4)

where Ω_E_ is the modulus of the Earth rotation vector, ψ is the astronomic latitude at the position where the experiment is performed on the site of Paris Observatory, and Θ*_N_* is the angle of the rotation stage corresponding to geographical north.

We realize two independent measurements with Raman beams oriented in the *X* and *Y* directions, i.e., with interferometer areas perpendicular to each other (Fig. 1). The two interferometers operate on the same physical principle (Eq. 3) but with different scale factor and bias term, thus increasing our confidence in the final result.

The phase shift measured at the output of the atom interferometer, Φ, is dominated by the rotation-induced Sagnac term of interest (of the order of 200 rad) and contains other bias terms on the order of a few tens of mrad detailed in Materials and Methods.

**Fig. 2.**
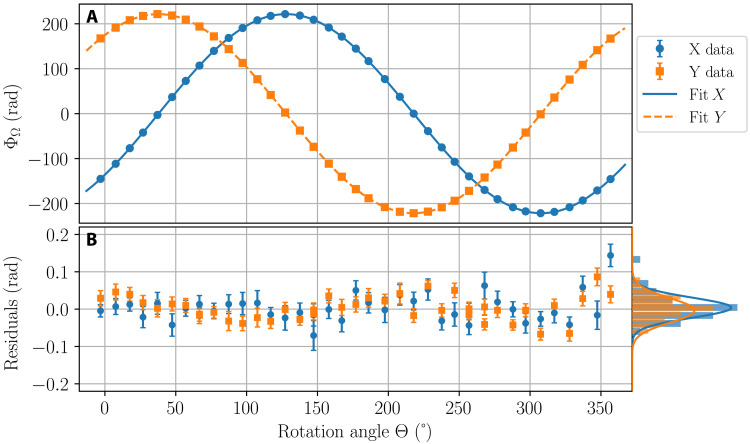
Measurement of the Sagnac phase shift with the two-axis atom interferometer. (**A**) Phase shift acquired for the *X* (blue dots) and *Y* (orange squares) directions as a function of the rotation angle Θ. Each point is a mean of typically 1500 realizations, with statistical error smaller than the symbol size. Lines are the least-squares fits with Eq. (5). (**B**) Difference between the data and the fits for *X* (blue dots) and *Y* (orange squares). Statistical error of each point is on the order of 30 mrad, and the histogram of the residuals (projected on the right side) has an SD of 40 mrad.

Figure 2 shows a typical measurement of the phase shift of the atom interferometer for both directions, acquired during 1 week in April 2021. Despite the interferometer measuring a phase shift modulo 2π in a given orientation, the complete 360° variation of the rotation angle allows us to unambiguously “unfold” the full ∼200 rad dephasing (see the Supplementary Materials). The data are fitted with

$$\Phi_{\Omega }^{x,y}(\Theta )=\Phi_{0}^{x,y}\text{cos}\;(\Theta -\Theta_{N}^{x,y})+B^{x,y}$$ (5)

where $\Phi_{0}^{x,y}$, $\Theta_{N}^{x,y}$, and *B*^*x*,*y*^ are free parameters (three for each direction). We extract $\Phi_{0}^{x}=221.572(9)$ rad and $\Phi_{0}^{y}=221.545(9)$ rad with fit residuals characterized by histograms with Gaussian width of about 40 mrad, comparable with the error bar of the individual points. Additional deviation in fit residuals can be explained by slow drifts of the bias during the measurement (see the Supplementary Materials). The extracted relative angular mismatch of the *X* and *Y* directions from being perfectly orthogonal is found to be 0.7(1) mrad, compatible with mechanical tolerance on the orthogonality of the sides of the vacuum chamber.

We now estimate the gyroscope scale factor for both directions (*X* and *Y*), i.e., evaluate the parameters entering Eq. 4. As we will show, all the parameters can be determined solely by frequency (or time) measurements, i.e., with high accuracy.

We measured the angle $\theta_{0}^{x,y}$ with the four-pulse interferometer by exploiting its residual sensitivity to continuous accelerations (see the Supplementary Materials) and obtained $\theta_{0}^{x}$ = 4.0750(5)° and $\theta_{0}^{y}$ = 4.1251(3)°. The interrogation time is derived from the clock of the experimental control system that is referenced to a highly stable and reproducible frequency standard. To check for small possible systematic deviations, we measure the time interval between the Raman pulses with a high-speed oscilloscope and find a value *T* = 400.0020(1) ms, with error bar limited by available temporal resolution. The local gravity acceleration value *g* has been previously measured in the laboratory using a transportable cold atom gravimeter. Since the value of *g* was affected by tides during the present measurement campaign, we take the maximal annual tide-induced variation of 3 × 10^−6^ m s^−2^ as an upper bound for the uncertainty on the value of *g*.

At the level of accuracy of typically 50 ppm (as demonstrated by the presented single dataset), we must account for the fact that the modulus of the wave vectors for the bottom [$k_{\text{eff}}^{\left(B\right)}$] and top [$k_{\text{eff}}^{\left(T\right)}$] Raman beams might differ by $\Delta k_{\text{eff}}\equiv k_{\text{eff}}^{\left(B\right)}-k_{\text{eff}}^{\left(T\right)}$, which introduces a correction to Eq. 4 at first order in ϵ = Δ*k*_eff_/*k*_eff_ (*31*).

We measure the values of ϵ^*x*, *y*^ via an interferometric measurement as explained in the Supplementary Materials, leading to ϵ*^x^* = 0.7(9) × 10^−6^ and ϵ*^y^* = 6.0(9) × 10^−6^.

We evaluate the astronomical latitude ψ—the angle between the local vertical (i.e., the vector perpendicular to the geoid) and the equatorial plane. It differs from the geographic latitude by the vertical north deflection, which can reach several arc seconds in regions where the geoid deviates noticeably from the ellipsoid of reference (e.g., close to mountains). Our experiment is positioned in a room of the Paris Observatory, at the geographic latitude of 48.83561(2)°. The vertical deflection was estimated to +0.95(4)″, yielding ψ = 48.83587(3)°.

Second order (∝Ω^2^), recoil [$\propto \hbar^{2}k_{\text{eff}}^{2}/(2m)$], and other residual terms appear in the expression of the Sagnac phase shift when the full calculation is developed (see the Supplementary Materials). These contributions would correspond to a relative correction of a few 10^−7^ to the estimation of the scale factor, which is two orders of magnitude below the accuracy of our measurement and have thus been neglected in this study.

Table 1 summarizes the measurements of the parameters for both directions. On the basis of these measurements, we estimate the theoretical values for the Sagnac phase shift as $\Phi_{\text{theo}}^{x}=221.5702(3)$ rad and $\Phi_{\text{theo}}^{y}=221.5574(2)$ rad.

**Table 1.** Error budget for the determination of theoretical Sagnac phase shift. The table lists the parameters entering the scale factor of the cold atom gyroscope. The right column is the uncertainty on the scale factor resulting from error propagation on each parameters.

**Parameter**	***X***	***Y***	**Relative** **uncertainty** **(ppm)**
*k*_eff_	14743247.08(4) m^−1^		0.003
*T*	400.0020(1) ms		0.75
*g*	9.809279(3) m s^−2^		0.3
θ_0_	4.0750(5)°	4.1251(3)°	0.6∣0.4
ϵ	−1.7(1) × 10^−6^	−9.3(2) × 10^−6^	0.07∣0.13
ψ	48.83587(3)°		0.6
Ω*_E_*	7.2921150(1) × 10^−5^ rad s^−1^		0.01
**Theoretical** **Sagnac phase**	221.5702(3) rad	221.5574(2) rad	1.2∣1.1

**Fig. 3.**
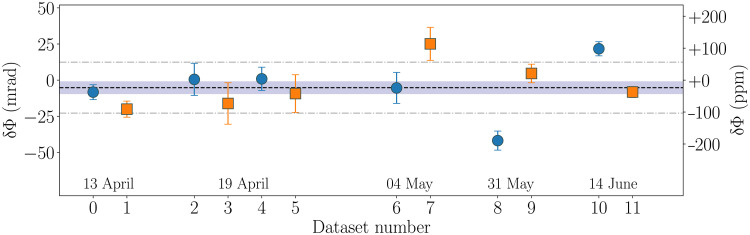
Comparison between experiment and theory. The data points represent the difference between the measured gyroscope phase shift and the theoretical Sagnac phase shift. Blue points (orange squares) are the data for the *X* (*Y*) axis. The dashed line represents the mean value of all the data (*X* and *Y*), and the shaded region is the standard error on the mean. Point-dashed lines indicates the SD on the set of measurement. The week of dataset acquisition (during year 2021) is indicated at the bottom along with the dataset number.

To reinforce the overall confidence in our measurements, we acquired six full-turn data from April to June 2021 and with different experimental parameters (e.g., variation of interrogation time *T*). We applied identical data treatment and fitting procedures (as described above) to all datasets and extracted corresponding fit parameters $\Phi_{0}^{x,y}$ (see the Supplementary Materials for the raw data and full fit results). In Fig. 3, we present the differences between the measured values and the corresponding theoretical expectations, $\delta \Phi^{x,y}=\Phi_{0}^{x,y}-\Phi_{\text{theo}}^{x,y}$.

The results have an overall good agreement, with a mean value close to zero (horizontal dashed line) and a standard error on the mean covering a substantial part of the data (gray-shaded region). The dispersion between the measurements is not fully captured by the uncertainties in fitted values of $\Phi_{0}^{x,y}$ of the corresponding datasets. We show in the Supplementary Materials with additional simulations that the deviations are consistent with a residual shift of the bias during the week-long measurements necessary to rotate the experiment. In conclusion, the data are consistent with the Sagnac phase shift prediction within an uncertainty of 25 ppm, dominated by the statistical uncertainty.

## DISCUSSION

The fundamental interest in our cold atom interferometer relies on its ability to measure several components of the local angular velocity and to explore rotational signals along different directions, in contrast to large ring laser gyroscope infrastructures where the gyroscope axes are fixed. This allows search for smaller signals beyond Earth’s rotation: In addition to local angular velocities induced by geological origins, one can constrain astrophysical rotations (orbit in the solar system, rotation in the Galaxy) or even rotations related to the fundamental structure of the universe. As shown in (*21*), Gödel’s model of the universe predicts a global rotation rate inducing a Sagnac phase shift. The mass density of the universe inferred from the 2018 Planck mission data (*32*) corresponds to a rotation rate of the order 10^−19^ rad s^−1^, far beyond the accuracy of current gyroscopes. However, the accuracy of our experiment gives an upper limit on the Gödel’s rotation obtained for matter waves (instead of photons in the case of the Planck mission) in a local measurement. From another point of view, the ability to detect signals at different frequencies in the experiment is a powerful tool to explore violation of Lorentz invariance. Following (*33*), our experiment might put constraints on parameters of alternative theories such as the standard model extension.

In addition, the precise knowledge of the scale factor of our gyroscope together with that of Earth’s rotation rate allows us to perform measurements of the vertical deflection at the level of few arc seconds. This provides a measure of the local gravity direction that depends on local mass anomalies. Accurate knowledge of the vertical deflection (which can amount to angles of a few arc seconds in flat areas and up to 50^′′^ in mountainous terrain) is widely used in geodesy and for geophysical purposes. A high-accuracy gyroscope such as ours allows to measure at least the north-south component of the vertical deflection in the zones where astronomical determination is impossible, e.g., for geodesy and geographical positioning in underground facilities.

Our work paves the way toward applications in rotational seismology, a field that studies rotational motions induced by earthquakes, explosions, and ambient vibrations (*34*), of interest for the understanding of the underground structure (*35*) or seismic hazard assessment in civil engineering (*36*). Theoretical studies have shown the benefit of using precision rotational sensing to improve the characterization of earthquake sources (*37*) and their localization (*38*)—the information of prime importance for seismic alert systems.

Accurate assessment of ground rotational signals is also of prime importance in the development of ground-based gravitational wave detectors, which is expected to be limited at low frequencies (below 1 Hz) by Newtonian noise (*39*, *40*) and rotational ground motion (*41*). Measuring and compensating these effects requires highly sensitive and accurate rotational sensors on the level of performances of our instrument (*42*).

These measurements at geophysical sites of interest require transportable gyroscopes with scale factors that are stable over weeks and are known with high accuracy (better than 100 ppm). While fiber optic gyroscopes (*43*) have been particularly developed and deployed for rotational seismology applications, reaching such stability and accuracy levels is challenging. Our cold atom sensor could lead to a transportable laboratory instrument (*44*) or even to an industrial product with increased robustness against environmental instabilities (temperature, vibrations, etc.), as achieved for cold atom gravimeters (*45*). A specific effort should address the control of the bias drift of our gyroscope because of the fluctuations of atomic trajectory coupled to relative mirror misalignment (*46*), which appeared as a limiting factor in the present work (see the Supplementary Materials). Provided with such proper engineering, the level of accuracy reported by our work opens a field of applications, with major scientific and societal impacts.

## MATERIALS AND METHODS

### Preparation and detection of the atoms

Cesium atoms laser cooled to a temperature of 1.8 μK are launched vertically in a fountain configuration at a velocity of 5 m s^−1^. After a quantum state selection in the least sensitive magnetic sublevel ∣*m_F_* = 0〉, the atoms enter the light-pulse interferometer, where a sequence of stimulated two-photon Raman transitions split, deflect, and recombine the atomic de Broglie waves.

At the output of the interferometer, the phase difference between the two paths is inferred by measuring the internal state (entangled with the external state) populations of the atoms via fluorescence detection. We operate the interferometer in joint mode (*29*) such that the time of a full cycle equals the total interrogation time 2*T* ≃ 800 ms.

### Alignment of the interferometer

We use a dedicated alignment protocol (*46*) that allows setting the atomic launch velocity parallel to vertical (local $\vec{g}$) with an accuracy of typically 200 μrad. Once set, this alignment is preserved upon variation of the rotation angle during the full-turn acquisition by actively stabilizing the sensor’s tilt at the nrad level during the acquisition (see the Supplementary Materials).

### Phase shift of the interferometer

We write the total phase shift at the output of the interferometer as Φ = Φ_Ω_ + Φ_1_ + Φ_2_, where Φ_1_ and Φ_2_, respectively, encode ${\vec{k}}_{\text{eff}}$-independent and ${\vec{k}}_{\text{eff}}$-dependent bias phase shifts. The contribution of Φ_1_ is mostly due to the one-photon lightshift and is maintained below 10 mrad by alternating measurements every cycle between +*k*_eff_ and −*k*_eff_ and computing the half-difference between the data (see the Supplementary Materials).

The most important terms contributing to Φ_2_ are (i) a continuous accelerations–induced phase shift (*17*, *31*) and (ii) a phase shift associated with the imperfect alignment of the bottom and top mirrors retro-reflecting the Raman beams (see Fig. 1) coupled to imperfect launching of the atoms along gravity (*46*). We recall in the Supplementary Materials the origin of these phase shifts and explain the methods used to mitigate their contribution all along the measurements, for both the *X* and *Y* directions.

## Supplementary Materials

### This PDF file includes:

Sections S1 to S4 Figs. S1 to S8 Tables S1 to S3 References
