# Supplementary material for: Accurate measurement of the Sagnac effect for matter waves
Source: Sci Adv. 2022 Jun 10;8(23):eabn8009. doi: 10.1126/sciadv.abn8009 (PMC9187224; doi:10.1126/sciadv.abn8009)
Supplement: Supplementary file 1 — Sections S1 to S4 Figs. S1 to S8 Tables S1 to S3 References [file sciadv.abn8009_sm.pdf]

Science Advances
AAAS

Supplementary Materials for

Accurate measurement of the Sagnac effect for matter waves

Romain Gautier *et al.*

Corresponding author: Remi Geiger, remi.geiger@obspm.fr

Sci. Adv. **8**, eabn8009 (2022)
DOI: 10.1126/sciadv.abn8009

This PDF file includes:

Sections S1 to S4
Figs. S1 to S8
Tables S1 to S3
References

Section S1: Experiment details and analysis of data

Section S1.1: Single measurement

Extraction of Φ_Ω

Each phase measurement is extracted by alternating between two side of the interferometric fringe, in a way to be locked at the center and always stay in the linear range, subsequently getting rid of contrast and offset fluctuations.

Contributions from non-inertial phase-shifts Φ_1 are reduced by a factor 100 by alternating interleaved measurement of $+k_{\text{eff}}$ and $-k_{\text{eff}}$ momentum transfer.

Inertial phases shifts present in Φ_2 are dependent on the sign of k_{eff} and are the following:

First, a DC acceleration term originating from the asymmetry introduced on the 4 pulses geometry to prevents parasitic interferometers from recombining. The two middle π pulses are shifted by a quantity ΔT_a in the same direction (thus asymmetrically with respect to the apogee), producing a phase shift:

$$\Phi_{DC} = 2T\Delta T_a k_{\text{eff}}(g \sin\theta_0 - \alpha)$$

where ΔT_a is the asymmetric timeshift introduced, $g\sin\theta_0$ is the projection of the gravity on the Raman beams, and α represents the rate of the frequency ramp applied on the Raman lasers to compensate for the variation in Doppler effect during the intereferometer. The ramp is first adjusted in a way to cancel the effect to a large extent, from 200 rad to hundreds of mrad.

To reject it even more, we alternate measurements of $+\Delta T_a$ and $-\Delta T_a$ to remove any residual sensitivity to DC acceleration.

The final phase is extracted from both the alternation of the sign of $\vec{k}_{\text{eff}}$ and ΔT_a as follow :

$$\Phi_\Omega = \frac{1}{2}\left[\left(\frac{\phi^{+k}_{+\Delta T_a} - \phi^{-k}_{+\Delta T_a}}{2}\right) + \left(\frac{\phi^{+k}_{-\Delta T_a} - \phi^{-k}_{-\Delta T_a}}{2}\right)\right]$$

The non inertial terms will appears in the half sum of the $+k_{\text{eff}}$ and $-k_{\text{eff}}$ signals and the terms from the DC acceleration appears in the half difference of $+\Delta T_a$ and $-\Delta T_a$ signals for opposite k_{eff} signals. Examples of the useful and rejected signals are presented in figure S1.

Alignement of the Raman beams

Another inertial signal which need to be reduced is due to the coupling between the alignment of the two Raman mirrors and the trajectory of the atoms (it appears if atoms are not launched perfectly along the gravity acceleration vector).

By repeating the procedure described in (46) we are able to align the two mirrors at the level of 3μrad, and adjust the vertical and transverse velocity with an accuracy of 1mm.s^{-1}, thus limiting this systematic effect at the beginning of the measurements at the level of 12 mrad.

Vibrations

To prevent the intereferometric signal from being impaired by the vibrations, the noise is reduced by hybridizing our atomic interferometer with two classical sensors (30). The two seismometers (3 Axis Trillium Compact 100 Hz, 120s bandwidth) are rigidly fixed to the Raman retro-reflection mirrors of the X axis and the vacuum chamber, and are used to record the vibrations during the interferometer. The three axis sensors allow us to extract vibration both for the X and Y measurements.

The signal measured by the two seismometers are combined with the transfer function of the experiment to estimate the phase shift due to vibrations (30, 47), which is fed-back to the Raman lasers in real time to be compensated. This reduce the vibration noise contribution to around 300 mrad phase noise per shot, equivalent to a rotation stability of 7×10^{-8} rad.s$^{-1}/\sqrt{\tau}$.

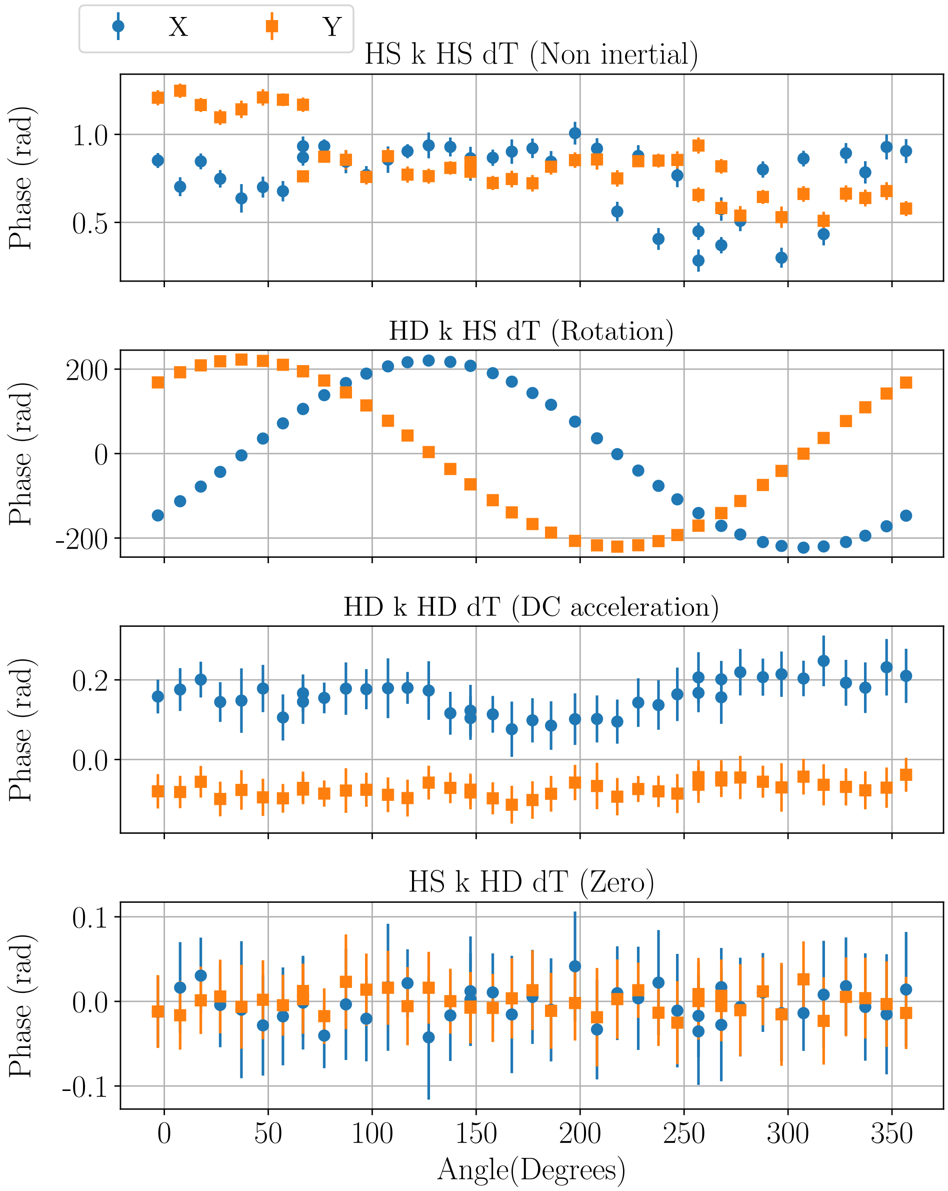

Figure S1: **Separated signals for a single dataset for the two axis.** The dataset correspond to the one presented on Figure 2 from the main text. From the different combinations of signals, we can extract multiple phase information. From the half sum of k_{eff} and the half difference of ΔT_a comes the non inertial terms. In the half difference of k_{eff} and the half sum of ΔT_a comes the Sagnac phase shift. In the half difference of k_{eff} and the half difference of ΔT_a comes the DC acceleration. From the half sum of k_{eff} and the half difference of ΔT_a, no terms should be left, leaving a constant zero phase shift.

Section S1.2: Control of the rotation angle

The whole experiment is mounted on a floating platform (Minus-K) which is itself mounted on a rotation stage (ALAR-250LP by Aerotech), and can be rotated by 360° with an accuracy of 10 $\mu\mathrm{rad}$. The rotation platform is not automatized and it's electronics used only for a readout of the relative angle of the experiment Θ.

When the experiment is rotated, it can happen that the angle between the rotation stage and the vibration isolation platform differs by a non negligible amount due to some twist or cables pulling on the experiment (it can reach up to $\pm 5\mathrm{mrad}$) .

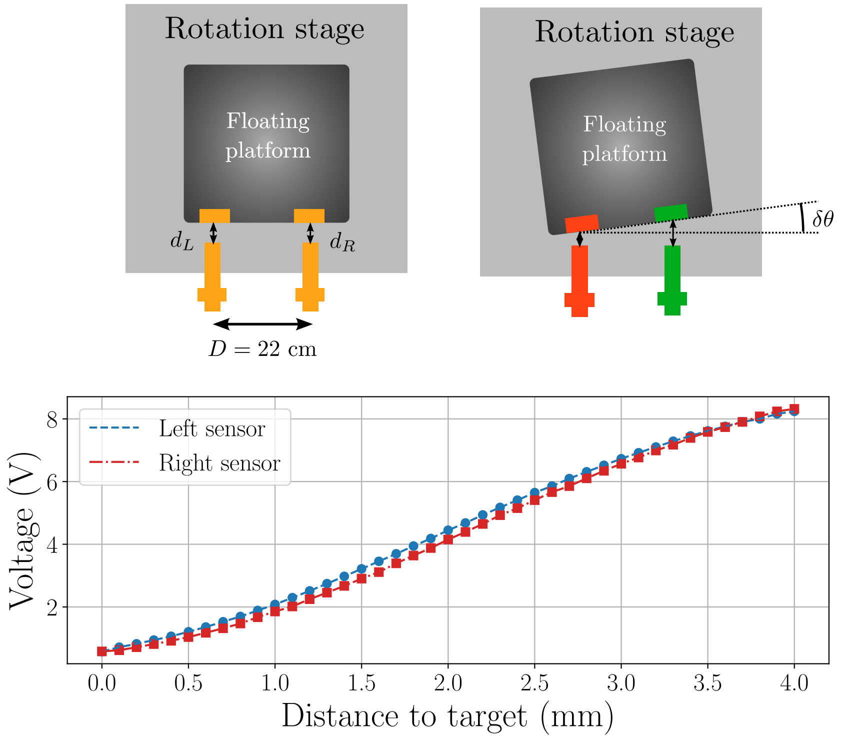

Figure S2: **Schematics of the rotation stage, the floating platform, and the two position sensors used to estimate the relative rotation $\delta\theta$ between the stage and the platform.** Bottom figure presents the characterization of the two proximity sensors, which have a slightly different response. From the voltage measured by the two sensors, we can deduce the angle between the rotation stage and the floating platform with 10 μrad accuracy.

To measure the angle between the two platforms, we installed two inductive proximity sen-

sors (Contrinex DW-AS-509-M12-390) separated by a baseline of 22.0(1) cm. The two sensors are fixed on the rotation stage, while the two steel target blocks are fixed in front, on the floating platform. By reading the voltage of the two sensors and using the calibration curve showed in Fig S2, we can deduce the respective distance between the sensors and the platform, allowing us to measure the angle by which the experiment effectively rotated, with $10\ \mu$rad accuracy.

Section S1.3: Details on one data-set

For each orientation of the experiment, we integrate the measurement over 20 min, once for the X axis, and once for the Y axis. Error bars on each points corresponds to the standard deviation on the measurement.

Once we have a measurement for the two axis for a given orientation of the experiment, we change the orientation by steps of 10 or 20 degrees, or smaller depending on the dataset.

In order to prevent, as much as possible, the impact of a potential drift of the gyroscope bias on the quality of a dataset, the experiment was not rotated in one single turn from 0° to 360°. Instead, for example, we rotated the experiment in one way from 0° to 360° by steps of 20°, and back from 350° to 10° by steps of 20° (i.e. interleaved with respect to the first way), leading effectively to an angular increment of 10°. Such a procedure reduces the impact of correlated noises on the measurement, while keeping the number of effective rotation of the experiment lower than if the rotation angles were randomly selected.

As the amplitude of variation of the measured phase is much greater than 2π, at first, only the top of the sinusoid is used to fit the data where we can stay in a 2π range. The following data-points are adjusted with an even number of π automatically added to match the first fit.

Section S1.4: Details on the full datasets

Six distinct datasets have been taken both for X and Y axes. Each full measurement for the two axes takes 1 week, with around 10 rotation of the apparatus per day. The extracted amplitude, phase and offset are presented on the table below.

For the datasets 2, 3, 4 and 5, instead of taking one measurement for X and one measurement for Y, we took alternatively a measurement for X with 2T = 800ms, a measurement for X with 2T = 801 ms and the same for Y. To be able to complete the measurements in one week, we did only half the rotation angles for each datasets.

Axis	Dataset #	2T (ms)	$\Delta T_a (\mu s)$	NPoints	Φ_0 (rad)	Θ_N (rad)	B (rad)	χ^2_{red}
X	0	800	60	41	221.572(9)	0.908341(41)	0.787(6)	3.9
	2	800	60	24	221.572(16)	0.908015(73)	1.004(12)	5.0
	4	801	60	24	222.409(12)	0.908125(57)	0.979(8)	3.3
	6	800	40	42	221.569(16)	0.907845(72)	0.731(11)	11.6
	8	800	60	46	221.524(9)	0.908029(43)	0.833(7)	2.6
	10	800	60	41	221.587(7)	0.909072(40)	0.972(6)	2.7
Y	1	800	60	41	221.545(9)	-0.661596(39)	0.628(6)	3.2
	3	800	60	24	221.548(23)	-0.662011(95)	0.678(15)	14.3
	5	801	60	24	222.389(20)	-0.662023(83)	0.665(14)	12.3
	7	800	40	41	221.584(16)	-0.662582(74)	0.690(11)	13.2
	9	800	60	43	221.557(11)	-0.662044(51)	0.545(8)	6.3
	12	800	60	41	221.559(7)	-0.660894(31)	1.238(5)	2.5

Table T1: **Detail of the 6 datasets taken for the two axis with the fitted parameters and their extracted uncertainties.** Are presented the parameters for each measurements including the total interrogation time 2T, the time asymetry used to prevent the parasitic interferometers from recombining ΔT_a, the number of points per datasets N, the fitted values of the control parameters Φ_0, Θ_N, and B and the corresponding reduced chi-squared for the least-square fit.

In the table T1 are presented the details and the results for the fitted parameters for all 12 measurements. The parameters that have been varied between datasets are T, ΔT_a and

the number of points per sets. The fitted parameters corresponds to Φ_0 (the amplitude of the sine), Θ_N the angle where the interferometer is pointing North, and the offset B. Next to this is presented the reduced chi-squared, as an indicator of the quality of the fit.

The angle between the two axes of the gyroscope is found stable at $89.9599(94)^\circ$, which represents a deviation of 0.7 mrad with respect to 90° (consistent with mechanical tolerance).

Section S2: Scale factor estimation

Measurement of T

Measurement of the duration of the interferometer has been done with a high resolution oscilloscope (Textronix 4 series Mixed signal oscilloscope) measuring directly the time between the Raman pulses photodiodes signals and yields a result of 400.0020(1) ms.

Measurement of g

Measurement of g has been performed at the position of the experiment with a cold atom gravimeter (CAG). Value has been corrected from the vertical gradient, accounting for an experiment at 1 m height.

Measurement of θ_0

To lift the degeneracy between $+k_{\text{eff}}$ and $-k_{\text{eff}}$, the Raman beams are tilted by an angle θ_0. The accurate measurement of this angle is necessary to estimate the expected scale factor value of the gyroscope.

One way to estimate the angle is to keep the 4 pulses geometry and change both the time asymmetry ΔT_a and the rate of the Doppler compensating ramp α. The ramp is necessary to maintain the resonance condition while the atoms are moving, as the Doppler detuning rise linearly. Expected phase shift is

$$\Phi = 2T\Delta T_a \left(k_{\text{eff}} g \sin(\theta_0) - \alpha\right) \tag{1}$$

By finding the ramp that cancel the phase shift, one can extract the angle of the Raman beams, in the same way that compensating the ramp is used to determine g on the cold atom gravimeters.

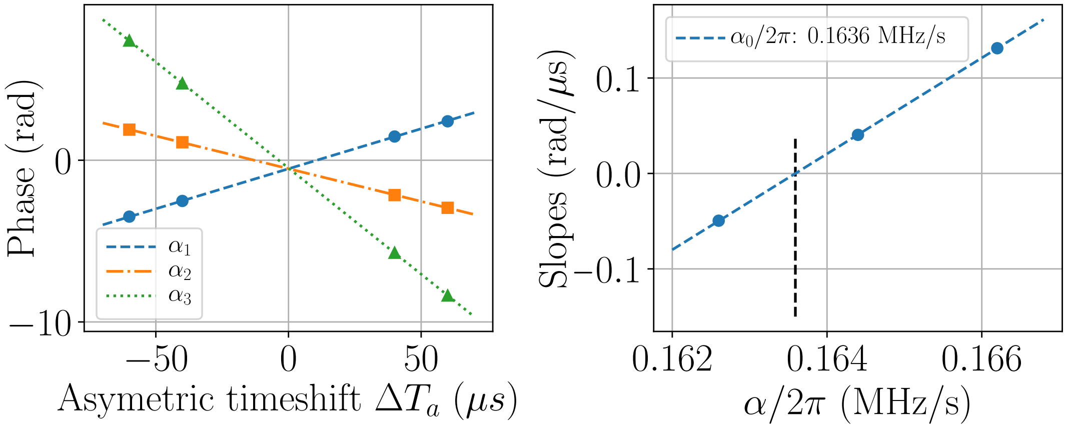

Figure S3: **Measurements of the angle of the Raman collimators.** On the left is presented the phase shifts versus the asymmetric time shift for 3 different ramp rate. On the right is the slopes of the previous curves versus the ramp rate applied. The Ramp where the slope is 0 is the one perfectly compensating the Doppler effect and is used to calculate the angle.

The measurements yields the following results: $\theta_0^x = 4.0750(5)°$, and $\theta_0^y = 4.1251(3)°$.

Measurement of Δk_{eff}

As studied in (32), a different angle between the two Raman collimators lead to a different modulus of the k_{eff} vector of both Raman beams, as illustrated on figure S4.

This Δk_{eff} between the two beams is defined as $k_{\text{eff}}^{(B)} - k_{\text{eff}}^{(T)}$, where $k_{\text{eff}}^{(B)}$ represents the wave vector of the bottom beam and $k_{\text{eff}}^{(T)}$ the one from the top beam. This Δk_{eff} introduce a correction to the scale factor of the experiment and prevents the interferometer from closing correctly.

In presence of a Δk_{eff} and for a 4 pulses interferometer, the two middle pulses needs to be shifted symmetrically by ΔT_s with respect to the apogee to close the interferometer. If the

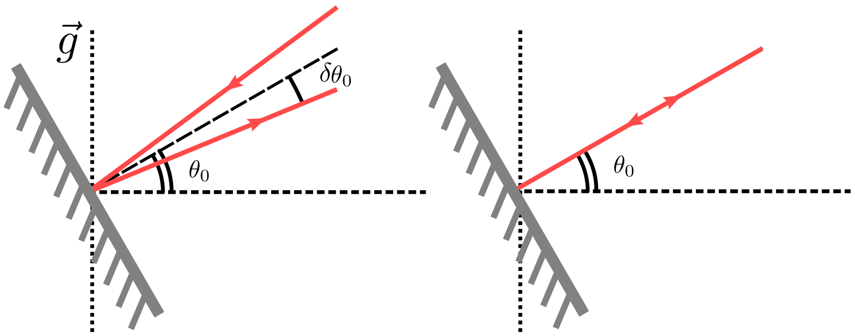

Figure S4: **Illustration of the effect of a misalignment of one beam on the modulus of k_{eff}.** Side by side are presented a misaligned wave vector and a perfectly aligned one.

closing condition:

$$\Delta T_s \simeq \frac{T\Delta k_{\text{eff}}}{2k_{\text{eff}}} \tag{2}$$

is not met, the maximum of contrast of the interferometer will be shifted in time. In a way to deduce the difference of k_{eff} between the two beams, we measure the contrast while varying the symmetric timeshift ΔT_s.

For the X axis, the measured shift is $-0.34(2)\mu$s and for the Y axis , the measured timeshift is $-1.87(4)\mu$s, corresponding respectively to a Δk_{eff} of $-25(1)m^{-1}$ for X and $-138(3)m^{-1}$ for Y. The origin behind the non negligible shift in the Y axis is of a technical nature, the Raman collimators for the X axis are outside the magnetic shield and the retro-reflection can be aligned by placing a target in front of it. The study of this effect and it's influence had been done on this easy to adjust axis (32). The Y collimators on the other hand are inside the two layers of magnetic shield and the misalignment cannot be easily corrected.

Presence of a Δk_{eff} will introduce a correction to the scale factor of the gyroscope associated with an imperfect recombination of the arms of interferometer. It is possible to reduce the corrections by adjusting the timings of the central π pulses with a ΔT_s to be at the maximum of contrast, in the same way that we measured it previously with the 4 pulses interferometer.

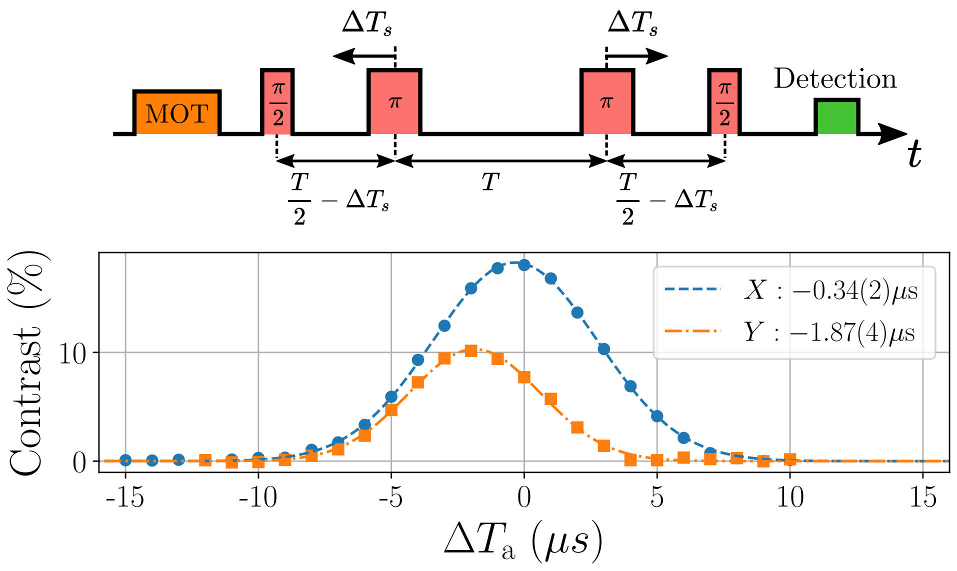

Figure S5: **Illustration of the sequence used to measure the Δk_{eff} with the 4 pulses interferometer.** Contrast for X and Y axis (respectively blue dots and orange squared points) versus the displacement of the two central pulses by ΔT_s. Blue dashed (orange point-dashed) lines are Gaussian fits to the data.

Once the timings are adjusted and taking into account the uncertainty on the value of ϵ, the full scale factor becomes:

$$\Phi_\Omega(\Theta) = \frac{T^3}{2} k_{\text{eff}} (1 - \frac{2}{3}\epsilon - \frac{11}{3}\delta\epsilon) g \cos(\theta_0) \times \cos(\psi)\Omega_E \times \cos(\Theta - \Theta_N) \tag{3}$$

where $\epsilon = \Delta k_{\text{eff}}/k_{\text{eff}}$ is the measured value for the misalignment of the two beams, and $\delta\epsilon$ the uncertainty on this measurement.

For the X axis , ϵ is found to be $-1.7(1) \times 10^{-6}$ and $-9.3(2) \times 10^{-6}$ for Y. Respectively a relative contribution to the scale factor of 7.7×10^{-9}(0.07 ppm) for X and 4.2×10^{-8}(0.13 ppm) for Y.

Measurement of Ψ

We used GNSS data to find the geographic latitude our experiment is located at. This latitude is defined as perpendicular to the ellipsoid of reference which include both Earth's mass den-

sity model distribution and centrifugal acceleration parts. This geodetic latitude needs to be corrected to take into account local Earth inhomogeneities and altitude. Two corrections appears, the North deviation and the East deviation. However, we are only interested in the North deviation as it will change the projection of the Earth rotation vector on our experiment.

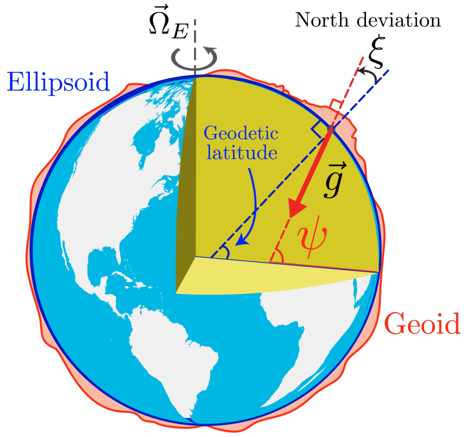

Figure S6: **Astronomical latitude.** Illustration of the Earth's geoid (in red) and ellipsoid (in blue). The geoid is the equipotential surface of the Earth's gravity field and the ellipsoid is the approximate shape of the Earth.

Calculations of the deviations have been done by the IGN Service de Géodésie et de Métrologie, and make use of the QGF16 quasi-geoid grid.

Section S3: Phase shift calculations and second order terms

The full calculation of the inertial phase shift yields other terms than the main Sagnac phase shift, mainly second order terms that are negligible at our level of sensitivity, but that would need to be considered if we were targeting ppm level measurements. The trajectories of the atoms have been calculated by solving the equation of motion taking gravity, Coriolis and centrifugal forces into account. The calculated positions and velocities are used to calculate the total phase shift at the output of the interferometer, with the timings and geometry of the experiment. From

the equation of motion is derived the position of the center of mass of the atoms at time t in the laboratory frame: $x(t)$, $y(t)$, and $z(t)$. From this the phase shift becomes:

$$\begin{aligned}\Phi &= \phi_1 - 2\phi_2 + 2\phi_3 - \phi_4 \\ &= \vec{k}_{\rm B}.\left[\vec{r}(0) - \vec{r}(2T)\right] + \vec{k}_{\rm T}.\left[-2\vec{r}\left(\frac{T}{2}\right) + 2\vec{r}\left(\frac{3T}{2}\right)\right]\end{aligned} \tag{4}$$

where the two effective wave vectors for the top beam ($k_{\rm T}$) and the bottom beam ($k_{\rm B}$) are distinguished as to take into account the possible effects on the trajectory if they are found not to be equal.

The phase shift has been developed in order of Ω and the terms are presented in table T3, with their relative values compared to the main Sagnac phase shift (In the table with a contribution of 1).

Secondary terms include:*(i)* coupling between inaccurate trajectory (v_{x0}, v_{y0} and v_{z0}) and the residual $k_{\rm eff}$ misalignment represented by ϵ; *(ii)* coupling between the introduced time asymmetry ΔT_a and ϵ; *(iii)* Second order terms in ΔT_a, *(iv)* second order terms in Ω^2 coupled with ΔT_a and finally *(v)* recoil terms that appear by taking the recoil of the atoms into account for their trajectories. Note that in the perfect case of no time asymmetry ΔT_a and a zero misalignment between the wave-vectors, only the Sagnac phase shift and the recoil terms will remain.

One of the second order term is the one due to ΔT_a which is not compensated by alternating $\pm\Delta T_a$ measurements:

$$2T\Delta T_a^2 k_{\rm eff}\, g\Omega\cos(\psi)\cos(\theta_0)\sin(\theta_N)$$

This term has a relative contribution of 9×10^{-8} for a typical ΔT_a of $60\mu s$.

Contributions from the finite pulse duration needs to be taken into account at the level of few 10^{-9} or below, and are not presented here.

Fluctuation of the modulus of g due to the tides can modify the scale factor at the level of 3×10^{-7}, but can be taken into account with an accurate tide model like it is done for gravity

measurements.

Terms due to vertical and/or horizontal gravity gradient can appear, but at the level of few 10^{-7}.

Fluctuations of the position of the geographical North, principally the Chandler and annula wobble have been estimated in (48) and account for 20 to 100 of p-rad.s^{-1}, below the current accuracy of our instrument.

Below is presented a table recapitulating the second order terms and their respective contribution to the total phase shift. All of those term can be safely neglected at the level of accuracy of our experiment.

Terms independent of Θ (Bias terms)	Absolute Phase value (rad)
$-2\, k_{\text{eff}}\, g\, T \sin(\theta_0)\, \Delta T_a$	-5.1×10^{2}
$+2\, k_{\text{eff}}\, g\, T \sin(\theta_0)\, \Delta T_a\, \epsilon$	4.9×10^{-5}
$-2\, k_{\text{eff}}\, T\, \cos(\theta_0)\, \epsilon\, v_{x_0}$	-1.2×10^{-1}
$+2\, k_{\text{eff}}\, T\, \sin(\theta_0)\, \epsilon\, v_{z_0}$	8.4×10^{-9}
$-4\, k_{\text{eff}}\, T^2\, \Omega\, \cos(\theta_0)\, \sin(\psi)\, \epsilon\, v_{y_0}$	-5.1×10^{-6}
$-4\, k_{\text{eff}}\, T\, \Omega\, \cos(\theta_0)\, \sin(\psi)\, \Delta T_a\, v_{y_0}$	-7.7×10^{-6}
$-\frac{\hbar}{m}\, k_{\text{eff}}^2\, T^3\, \Omega^2\, \cos(\theta_0)^2\, \cos(\psi)^2$	-1.5×10^{-5}
$+\frac{\hbar}{m}\, k_{\text{eff}}^2\, T^3\, \Omega^2\, (\cos(\theta_0)^2 + \cos(\psi)^2)$	5.1×10^{-5}

Table T2: **Bias terms coming from the full calculation of the Sagnac phase shift and their values.** Terms highlighted in gray rows are terms which will be canceled while alternating the sign of ΔT_a.

Section S4: Drift of the bias - Statistical simulation

During week long measurements, we can observe fluctuations of the alignment of the mirrors and of the atomic trajectories, creating a bias term drifting in time:

Terms proportional to Ω	Relative phase value
$-\frac{1}{2}\, k_{\text{eff}}\, g\, T^3\, \Omega\, \cos(\theta_0)\, \cos(\psi)\, \sin(\Theta)$	1
$+\frac{11}{6}\, k_{\text{eff}}\, g\, T^3\, \Omega\, \cos(\theta_0)\, \cos(\psi)\, \sin(\Theta)\, \epsilon$	3.7×10^{-5}
$+4\, k_{\text{eff}}\, T\, \Omega\, \cos(\theta_0)\, \cos(\psi)\, \sin(\Theta)\, \Delta T_a\, v_{z_0}$	3.1×10^{-7}
$+4\, k_{\text{eff}}\, T\, \Omega\, \sin(\theta_0)\, \cos(\psi)\, \cos(\Theta)\, \Delta T_a\, v_{y_0}$	2.2×10^{-8}
$+2\, k_{\text{eff}}\, T\, \Omega\, g\, \cos(\theta_0)\, \cos(\psi)\, \sin(\Theta)\, \Delta T_a^2$	9.0×10^{-8}
$+4\, k_{\text{eff}}\, T^2\, \Omega\, \cos(\theta_0)\, \cos(\psi)\, \sin(\Theta)\, \epsilon\, v_{z_0}$	2.0×10^{-7}
$+4\, k_{\text{eff}}\, T^2\, \Omega\, \sin(\theta_0)\, \cos(\psi)\, \sin(\Theta)\, \epsilon\, v_{x_0}$	1.5×10^{-9}
**Terms proportional to Ω^2**	
$-\frac{11}{3}\, g\, k_{\text{eff}}\, T^3\, \Omega^2\, \cos^2(\psi)\, \sin(\theta_0)\, \Delta T_a$	-1.5×10^{-9}
$+\frac{11}{3}\, g\, k_{\text{eff}}\, T^3\, \Omega^2\, \cos(\psi)\, \sin(\psi)\, \cos(\theta_0)\, \cos(\Theta)\, \Delta T_a$	2.2×10^{-8}
$-\frac{2\hbar}{m}\, k_{\text{eff}}^2\, T^3\, \Omega^2\, \sin(\theta_0)\, \cos(\theta_0)\, \cos(\psi)\, \cos(\Theta)$	-1.1×10^{-8}
$-\frac{\hbar}{m}\, k_{\text{eff}}^2\, T^3\, \Omega^2\, \cos(\theta_0)^2\, \cos(\psi)^2\, \cos(\Theta)^2$	-6.8×10^{-8}

Table T3: **First and second order terms coming from the full calculation of the Sagnac phase shift and their relative value compared to the first order approximation that is being used in the main text.** The terms appear to be coupling between the different asymmetries of the experiment, for example coupling between velocity errors (v_{x0}, v_{y0} and v_{z0}) and the asymmetric timing ΔT_a. We find as well couplings between ϵ due to the misalignment of the Raman beams and ΔT_a or velocity errors. If the full trajectories of the atoms are taken into account, appears some recoils terms both as bias phases and terms proportional to Ω^2. Higher order terms appears but only up to the second orders are presented here for clarity. Terms highlighted in gray rows are terms which will be canceled while alternating the sign of ΔT_a.

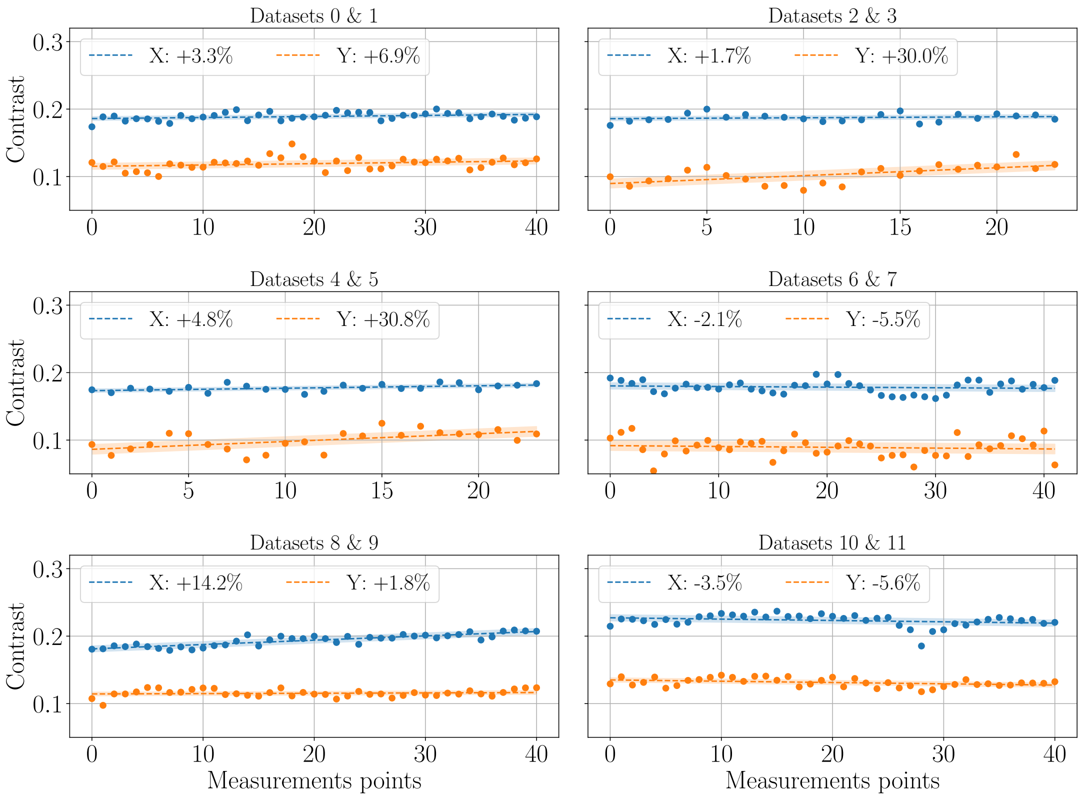

Figure S7: **Measures of the contrast for all datasets.** Each subplots presents the contrast for the X axis (Blue points, dashed blue line) and the Y axis (orange points, dashed orange lines), with a linear adjustment to estimate the drift during the measurement

$$\begin{aligned}\Phi(t) &= 2Tk_{\text{eff}}\Delta\theta(t)\Delta v(t) \\ &\approx 12\text{mrad}/\mu\text{rad}/\text{mm.s}^{-1}\end{aligned} \tag{5}$$

where $\Delta\theta(t)$ represents represents the mirror misalignment and $\Delta v(t)$ the error on the trajectory.

A visible consequence of mirror misalignment is a change on the contrast of the interferometer. Before any measurement point, we measure the contrast of the interferometer to have some insight on it's drift between measurements.

When we look at the contrast evolution for each datasets (See Fig S7) we can see long variations on the order of 5% to 30% , probably linked to temperature fluctuations of the laboratory

room impacting the mirror relative alignment. From this level of fluctuation we can deduce that the misalignment has to reach from 3 μrad to 9 μrad, knowing the evolution of the contrast with the misalignment (46).

On the other side, typical fluctuations of the launch velocity are of the order of σ_v = 1.0 mm.s^{-1} after alignment of the trajectories.

We use this estimation of the misalignment of both the mirror and the trajectories to simulate the impact of this effect on the measure of the Sagnac phase, which is on the order of 50 mrad. Datas are generated for experiment angles from 0 to 360 ° from a sine of amplitude equal to the expected Sagnac phase. The fluctuations of the bias are generated from the typical variations and then applied randomly as bias fluctuations. Results for the simulations are presented on figure S8 and are to be compared with the measured dataset.

The simulation show that a fluctuation of the bias during the measurements (due to the phase shift linked to the alignment of the mirrors and trajectory) can create a dispersion of the data bigger than the statistical uncertainty. Some simulated datasets can reach up to 50 mrad deviation with the parameters presented before, which could explain the deviation of some of our measured values (mostly the dataset 8, of the 31 May 2021, which is close to 50 mrad away from the expected value).

The level of misalignment and fluctuations of the trajectory needed to reproduce the effect is on the level that we can expect as well, showing that our observed dispersion can be explain by this effect.

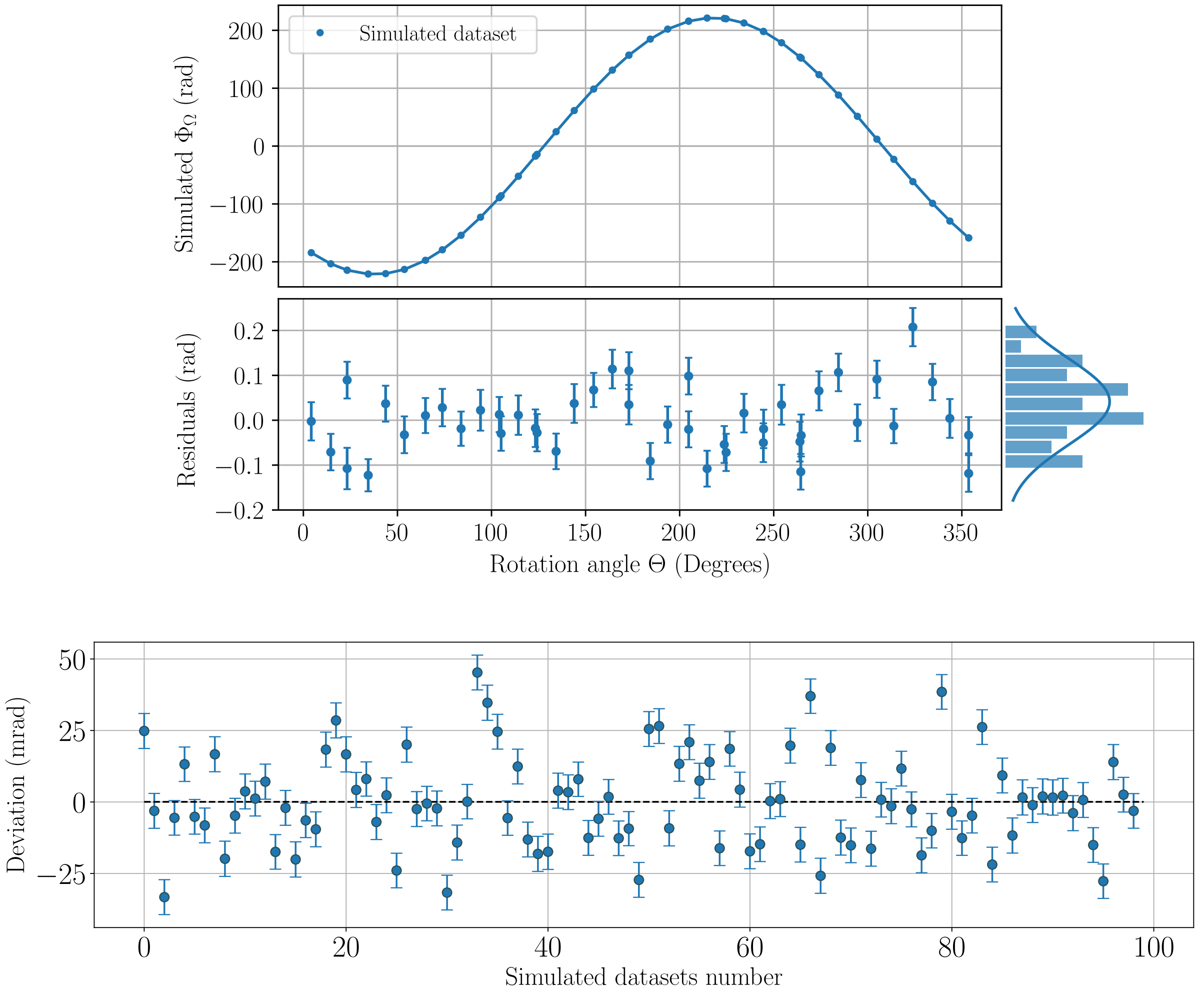

Figure S8: **Example of a simulated dataset and results of the simulation for 99 realizations.** Third subset represents deviation from the expected Sagnac phase for 99 simulated datasets, showing a dispersion bigger than the statistical noise, and with the order of magnitude matching the real case.

REFERENCES AND NOTES

1. G. Sagnac, L'éther lumineux démontré par l'effet du vent relatif d'éther dans un interféromètre en rotation uniforme. *C. R. Acad. Sci. (Paris)* **157**, 708–710 (1913).

2. E. J. Post, Sagnac effect. *Rev. Mod. Phys.* **39**, 475–493 (1967).

3. R. Anderson, H. R. Bilger, G. E. Stedman, "Sagnac" effect: A century of Earth-rotated interferometers. *Am. J. Phys.* **62**, 975–985 (1994).

4. A. A. Michelson and H. G. Gale, The effect of the Earth's rotation on the velocity of light, II. *ApJ* **61**, 140 (1925).

5. W. M. Macek, D. T. M. Davis Jr., Rotation rate sensing with traveling-wave ring lasers. *Appl. Phys. Lett.* **2**, 67–68 (1963).

6. V. Vali, R. W. Shorthill, Fiber ring interferometer. *Appl. Optics* **15**, 1099–1100 (1976).

7. H. C. Lefèvre, The fiber-optic gyroscope, a century after Sagnac's experiment: The ultimate rotation-sensing technology? *C. R. Phys.* **15**, 851–858 (2014).

8. J. E. Zimmerman and J. E. Mercereau, Compton wavelength of superconducting electrons. *Phys. Rev. Lett.* **14**, 887–888 (1965).

9. S. A. Werner, J.-L. Staudenmann, R. Colella, Effect of Earth's rotation on the quantum mechanical phase of the neutron. *Phys. Rev. Lett.* **42**, 1103–1106 (1979).

10. F. Riehle, T. Kisters, A. Witte, J. Helmcke, C. J. Bordé, Optical ramsey spectroscopy in a rotating frame: Sagnac effect in a matter-wave interferometer. *Phys. Rev. Lett.* **67**, 177–180 (1991).

11. F. Hasselbach and M. Nicklaus, Sagnac experiment with electrons: Observation of the rotational phase shift of electron waves in vacuum. *Phys. Rev. A* **48**, 143–151 (1993).

12. K. Schwab, N. Bruckner, R. E. Packard, Detection of the Earth's rotation using superfluid phase coherence. *Nature* **386**, 585–587 (1997).

13. R. W. Simmonds, A. Marchenkov, E. Hoskinson, J. C. Davis, R. E. Packard, Quantum interference of superfluid 3he. *Nature* **412**, 55–58 (2001).

14. K. C. Wright, R. B. Blakestad, C. J. Lobb, W. D. Phillips, G. K. Campbell, Driving phase slips in a superfluid atom circuit with a rotating weak link. *Phys. Rev. Lett.* **110**, 025302 (2013).

15. A. Lenef, T. Hammond, E. Smith, M. Chapman, R. Rubenstein, D. E. Pritchard, Rotation sensing with an atom interferometer. *Phys. Rev. Lett.* **78**, 760–763 (1997).

16. A. Gauguet, B. Canuel, T. Lévèque, W. Chaibi, A. Landragin, Characterization and limits of a cold-atom Sagnac interferometer. *Phys. Rev. A* **80**, 063604 (2009).

17. J. K. Stockton, K. Takase, M. A. Kasevich, Absolute geodetic rotation measurement using atom interferometry. *Phys. Rev. Lett.* **107**, 133001 (2011).

18. Y. Sato, Sagnac-based rotation sensing with superfluid helium quantum interference devices. *C. R. Phys.* **15**, 898–906 (2014).

19. B. Barrett, R. Geiger, I. Dutta, M. Meunier, B. Canuel, A. Gauguet, P. Bouyer, A. Landragin, The Sagnac effect: 20 years of development in matter-wave interferometry. *C. R. Phys.* **15**, 875–883 (2014).

20. R. Geiger, A. Landragin, S. Merlet, F. Pereira dos Santos, High-accuracy inertial measurements with cold-atom sensors. *AVS Quantum Sci.* **2**, 024702 (2020).

21. A. Delgado, W. P. Schleich, G. Süssmann, Quantum gyroscopes and Gödel's universe: Entanglement opens a new testing ground for cosmology. *New J. Phys.* **4**, 37–37 (2002).

22. A. Gebauer, M. Tercjak, K. U. Schreiber, H. Igel, J. Kodet, U. Hugentobler, J. Wassermann, F. Bernauer, C.-J. Lin, S. Donner, S. Egdorf, A. Simonelli, J.-P. R. Wells, Reconstruction of

the instantaneous earth rotation vector with sub-arcsecond resolution using a large scale ring laser array. *Phys. Rev. Lett.* **125**, 033605 (2020).

23. P. Berg, S. Abend, G. Tackmann, C. Schubert, E. Giese, W. P. Schleich, F. A. Narducci, W. Ertmer, E. M. Rasel, Composite-light-pulse technique for high-precision atom interferometry. *Phys. Rev. Lett.* **114**, 063002 (2015).

24. A. V. Rakholia, H. J. McGuinness, G. W. Biedermann, Dual-axis high-data-rate atom interferometer via cold ensemble exchange. *Phys. Rev. Appl.* **2**, 054012 (2014).

25. Z.-W. Yao, H.-H. Chen, S.-B. Lu, R.-B. Li, Z.-X. Lu, X.-L. Chen, G.-H. Yu, M. Jiang, C. Sun, W.-T. Ni, J. Wang, M.-S. Zhan, Self-alignment of a large-area dual-atom-interferometer gyroscope using parameter-decoupled phase-seeking calibrations. *Phys. Rev. A* **103**, 023319 (2021).

26. Y.-J. Chen, A. Hansen, G. W. Hoth, E. Ivanov, B. Pelle, J. Kitching, E. A. Donley, Single-source multiaxis cold-atom interferometer in a centimeter-scale cell. *Phys. Rev. Appl.* **12**, 014019 (2019).

27. C. L. G. Alzar, Compact chip-scale guided cold atom gyrometers for inertial navigation: Enabling technologies and design study. *AVS Quantum Sci.* **1**, 014702 (2019).

28. X. Wu, F. Zi, J. Dudley, R. J. Bilotta, P. Canoza, H. Müller, Multiaxis atom interferometry with a single-diode laser and a pyramidal magneto-optical trap. *Optica* **4**, 1545 (2017).

29. I. Dutta, D. Savoie, B. Fang, B. Venon, C. L. Garrido Alzar, R. Geiger, A. Landragin, Continuous cold-atom inertial sensor with 1 nrad/sec rotation stability. *Phys. Rev. Lett.* **116**, 183003 (2016).

30. D. Savoie, M. Altorio, B. Fang, L. A. Sidorenkov, R. Geiger, A. Landragin, Interleaved atom interferometry for high-sensitivity inertial measurements. *Sci. Adv.* **4**, eaau7948 (2018).

31. L. A. Sidorenkov, R. Gautier, M. Altorio, R. Geiger, A. Landragin, Tailoring multiloop atom interferometers with adjustable momentum transfer. *Phys. Rev. Lett.* **125**, 213201 (2020).

32. P. Collaboration, Planck 2018 results. *A&A* **641**, A4 2020.

33. S. Moseley, N. Scaramuzza, J. D. Tasson, M. L. Trostel, Lorentz violation and Sagnac gyroscopes. *Phys. Rev. D* **100**, 064031 (2019).

34. W. H. K. Lee, Rotational seismology, in *Encyclopedia of Natural Hazards*, P. T. Bobrowsky, Ed. (Springer Netherlands, 2013), pp. 877–879.

35. M. Bernauer, A. Fichtner, H. Igel, Inferring Earth structure from combined measurements of rotational and translational ground motions. *Geophysics* **74**, WCD41–WCD47 (2009).

36. D. Basu, A. S. Whittaker, M. C. Constantinou, Characterizing rotational components of earthquake ground motion using a surface distribution method and response of sample structures. *Eng. Struct.* **99**, 685–707, 2015.

37. S. Donner, M. Bernauer, H. Igel, Inversion for seismic moment tensors combining translational and rotational ground motions. *Geophys. J. Int.* **207**, 562–570 (2016).

38. Z. Li and M. van der Baan, Elastic passive source localization using rotational motion. *Geophys. J. Int.* **211**, 1206–1222 (2017).

39. J. Harms, E. L. Bonilla, M. W. Coughlin, J. Driggers, S. E. Dwyer, D. J. McManus, M. P. Ross, B. J. J. Slagmolen, K. Venkateswara, Observation of a potential future sensitivity limitation from ground motion at LIGO Hanford. *Phys. Rev. D* **101**, 102002 (2020).

40. B. Canuel, S. Abend, P. Amaro-Seoane, F. Badaracco, Q. Beaufils, A. Bertoldi, K. Bongs, P. Bouyer, C. Braxmaier, W. Chaibi, N. Christensen, F. Fitzek, G. Flouris, N. Gaaloul, S. Gaffet, C. L. G. Alzar, R. Geiger, S. Guellati-Khelifa, K. Hammerer, J. Harms, J. Hinderer, M. Holynski, J. Junca, S. Katsanevas, C. Klempt, C. Kozanitis, M. Krutzik, A. Landragin, I. L. Roche, B. Leykauf, Y.-H. Lien, S. Loriani, S. Merlet, M. Merzougui, M. Nofrarias, P. Papadakos, F. P. dos Santos, A. Peters, D. Plexousakis, M. Prevedelli, E. M. Rasel, Y. Rogister, S. Rosat, A. Roura, D. O. Sabulsky, V. Schkolnik, D. Schlippert, C. Schubert, L. Sidorenkov, J.-N. Siemß, C. F. Sopuerta, F. Sorrentino, C. Struckmann, G. M. Tino, G. Tsagkatakis, A. Viceré, W. von Klitzing, L. Woerner, X. Zou, ELGAR—A European

laboratory for gravitation and atom-interferometric research. *Class. Quantum Gravity* **37**, 225017 (2020).

41. M. P. Ross, K. Venkateswara, C. Mow-Lowry, S. Cooper, J. Warner, B. Lantz, J. Kissel, H. Radkins, T. Shaffer, R. Mittleman, A. Pele, J. Gundlach, Towards windproofing LIGO: Reducing the effect of wind-driven floor tilt by using rotation sensors in active seismic isolation. *arXiv*, 2003.06447 (2020).

42. B. Lantz, R. Schofield, B. O'Reilly, D. E. Clark, D. DeBra, Review: Requirements for a ground rotation sensor to improve advanced LIGO. *Bull. Seismol. Soc. Am.* **99**, 980–989 (2009).

43. F. Bernauer, J. Wassermann, F. Guattari, A. Frenois, A. Bigueur, A. Gaillot, E. de Toldi, D. Ponceau, U. Schreiber, H. Igel, Blueseis3a: Full characterization of a 3c broadband rotational seismometer. *Seismol. Res. Lett.* **89**, 620–629 (2018).

44. T. Farah, C. Guerlin, A. Landragin, P. Bouyer, S. Gaffet, F. P. dos Santos, S. Merlet, Underground operation at best sensitivity of the mobile LNE-SYRTE cold atom gravimeter. *Gyroscopy Navig.* **5**, 266–274 (2014).

45. V. Ménoret, P. Vermeulen, N. Le Moigne, S. Bonvalot, P. Bouyer, A. Landragin, B. Desruelle, Gravity measurements below 10^{-9} g with a transportable absolute quantum gravimeter. *Sci. Rep.* **8**, 12300 (2018).

46. M. Altorio, L. A. Sidorenkov, R. Gautier, D. Savoie, A. Landragin, R. Geiger, Accurate trajectory alignment in cold-atom interferometers with separated laser beams. *Phys. Rev. A* **101**, 033606 (2020).

47. P. Cheinet, B. Canuel, F. P. dos Santos, A. Gauguet, F. Yver-Leduc, A. Landragin, Measurement of the sensitivity function in a time-domain atomic interferometer. *IEEE Trans. Instrum. Meas.* **57**, 1141–1148 (2008).

48. K. U. Schreiber, T. Klügel, J.-P. R. Wells, R. B. Hurst, A. Gebauer, How to detect the Chandler and the annual wobble of the Earth with a large ring laser gyroscope. *Phys. Rev. Lett.* **107**, 173904 (2011).
